# “It’s my blood”: ethical complexities in the use, storage and export of biological samples: perspectives from South African research participants

**DOI:** 10.1186/1472-6939-15-4

**Published:** 2014-01-22

**Authors:** Keymanthri Moodley, Nomathemba Sibanda, Kelsey February, Theresa Rossouw

**Affiliations:** 1 Centre for Medical Ethics and Law, Department of Medicine, Faculty of Health Sciences, Stellenbosch University, Stellenbosch, South Africa; 2 School of Health Systems and Public Health, Faculty of Health Sciences, University of Pretoria, Pretoria, South Africa; 3 Centre for Medical Ethics and Law, Department of Medicine, Faculty of Health Sciences, Stellenbosch University, Stellenbosch, South Africa

## Abstract

**Background:**

The use of biological samples in research raises a number of ethical issues in relation to consent, storage, export, benefit sharing and re-use of samples. Participant perspectives have been explored in North America and Europe, with only a few studies reported in Africa. The amount of research being conducted in Africa is growing exponentially with volumes of biological samples being exported from the African continent. In order to investigate the perspectives of African research participants, we conducted a study at research sites in the Western Cape and Gauteng, South Africa.

**Methods:**

Data were collected using a semi-structured questionnaire that captured both quantitative and qualitative information at 6 research sites in South Africa. Interviews were conducted in English and Afrikaans. Data were analysed both quantitatively and qualitatively.

**Results:**

Our study indicates that while the majority of participants were supportive of providing samples for research, serious concerns were voiced about future use, benefit sharing and export of samples. While researchers view the provision of biosamples as a donation, participants believe that they still have ownership rights and are therefore in favour of benefit sharing. Almost half of the participants expressed a desire to be re-contacted for consent for future use of their samples. Interesting opinions were expressed with respect to export of samples.

**Conclusions:**

Eliciting participant perspectives is an important part of community engagement in research involving biological sample collection, export, storage and future use. A tiered consent process appears to be more acceptable to participants in this study. Eliciting opinions of researchers and research ethics committee (REC) members would contribute multiple perspectives. Further research is required to interrogate the concept of ownership and the consent process in research involving biological samples.

## Background

Biological sample availability creates important opportunities for researchers to advance medical science and contribute to collective good [1,2]. However several ethical, legal and social issues co-exist [3]. Research Ethics Committees (RECs) globally have a mandate to protect research participant interests such as confidentiality, ownership, export, storage and secondary use of samples (individual good) with specific consent, regulations and policies but implementation of these policies differs from one REC to another [4]. Researchers (unable to predict future use of samples due to scientific advances) prefer a broad general form of consent to advance scientific research and promote the collective good [1,5-9]. This divergence of perspectives creates tension between RECs that promote individual benefit and researchers who support collective benefit. Recently the tension has been exacerbated by participant groups instituting litigation for improper use of biospecimens - Havasupai Indian Tribe Case [10] and other groups requesting return of specimens - Yanamamo Tribe Case [11].

Consideration of best practices for use of biospecimens should begin with an exploration of participant expectations [10]. This is an important first step in discerning the complexity of the debate. Several studies have investigated participants’ preferences around the world - Egypt [12], Asia [9] Scotland [13], Sweden [14,15], Canada [2,16], United States [17], United Kingdom [18], Uganda [19] and the Netherlands [20]. In South Africa, however, such a process of public engagement has not occurred.

Debate should however be bidirectional with pathologists and researchers also actively engaged on consent for use of biological specimens. This process is important for fostering mutual understanding between researchers and participants and informing research ethics guidelines. Pathologists have an important contribution to make, as they are generally the custodians of biobanks and other stores of biological samples [8]. Pathologists and researchers share a common goal in terms of scientific research and the advancement of scientific knowledge [1], but hold a variety of views relating to the use of biological specimens [5-7,21].

There is generally strong support for broad consent as it is sometimes practically difficult to re-contact and re-consent participants [7,22]. Some believe that it is unethical not to store samples and conduct previously unforeseen future studies, and that, since research participants donate specimens, there is no need for external control on what research is conducted on them as society will ultimately benefit [22]. There is a school of thought that supports the use of research using biological samples donated to generate patents and profits that should not be shared with participants [23-25]. And of course, the contrary view is held as well [26].

Internationally RECs differ in terms of their consent requirements for use of biological samples and are criticised for their lack of consistency in policy development and enforcement [4]. In South Africa there is significant variance in the composition and functioning of RECs [27]. The existing 33 RECs registered with the National Health Research Ethics Council (NHREC) currently are not equally capacitated and hence differ in terms of their agendas, financing, resources and ethics capacity training.

Southern Africa is regarded as fertile ground for a wide range of research endeavours due to the enormous burden of infectious disease in the region, highly skilled medical researchers and large numbers of treatment naïve patients [28,29]. South Africa is positioned at the epicenter of the HIV pandemic and HIV research as well as HIV biobanking is flourishing scientifically. There are currently 1390 clinical trials registered on the South African Clinical Trials Register hosted by the Department of Health. Three years ago (2010) there were 946 trials registered [30]. This indicates a significant growth of clinical trial research over the past 3 years. South Africa (SA) is home to hundreds of research projects involving biospecimen collection, analysis, storage and future use. Exportation of samples to developed countries occurs frequently.

To date, apart from a small study conducted on 20 research participants at a single site, there has been very little empirical research published examining the ethical aspects of participant perspectives related to biospecimen use in research in SA [31]. This timely study has employed empirical research to elucidate perspectives on biospecimen use in research in SA with a view to informing guideline development, research ethics deliberation and legislation in this controversial research area. The Human Tissues Act No 65 of 1983 has been replaced by new legislation in the National Health Act No 61 of 2003 dealing with use of human tissue samples, biobanking, import and export of samples. There are gaps in some of this legislation with respect to export of samples, informed consent from participants and material transfer agreements. The National Health Research Ethics Council (NHREC) in SA is involved with the development of guidelines for the collection, use, storage and export of biological samples. These guidelines aim to address gaps in legislation and elaborate on ethical concerns more specifically. Findings from this study will therefore impact timeously on policy development.

We are aware of two reported research studies that used quantitative methods to investigate the perspectives of African research participants regarding sample storage and re-use, in Egypt [12] and Uganda [19]. The Ugandan study (n = 343) found that a large majority (95%) of participants would consent to their sample being re-used without additional consent, subject to approval by an Institutional Review Board (IRB). On the other hand, the study in Egypt (n = 600) found that just under half (44.3%) of the participants felt that consent forms should include a separate section relating to storage and future use of samples and data. To our knowledge, apart from a recent publication on biological sample use amongst 20 participants with Tuberculosis at a single study site in South Africa [31], a focus group study on biobanking in Nigeria amongst lay persons [32] and a broader study on consent for genetic and genomic research in Ghana [33], no other qualitative research studies have been reported in Africa. Considering the contextual complexity of these issues and the vast cultural and geographical diversity on the African continent, we believe it is of paramount importance to seek the views of South African research participants on these issues. Our study aimed to provide an exploration into these issues, specifically seeking to qualitatively explore the views of research participants on sample storage, export and future use.

## Methods

This study was conducted over a 10 month period from September 2011 to June 2012. We sampled 200 participants in the Western Cape and Gauteng (100 participants at each location) in order to capture as many different perspectives as possible, given the diversity of populations in South Africa. Research participants who had experience with research, the consent process and biological samples were recruited from academic research units attached to public hospitals and private research centres (6 research sites: 4 in the Western Cape and 2 in Pretoria, Gauteng). Principal investigators at the 6 clinical trial sites provided permission for the conduct of this sub-study. The participants had already provided consent to participate in the parent study. Investigators on the parent study then approached participants to inform them about our sub-study. Those who were interested in participating in the sub-study agreed to interviews. We re-consented the participants specifically for our biological samples sub-study. In total 200 semi- structured questionnaires were completed by research assistants during interviews in participants’ language of choice – with data recorded in English. Questionnaires included demographic details and study specific questions which focussed on perceptions of choice in providing samples, religious and cultural concerns related to biological samples, storage export and consent related to future use of samples. Explanations of biological samples and research ethics committees were provided before the related question was posed.

Interviews were conducted in English and Afrikaans by the same interviewer at each site. Interviews were recorded and transcribed verbatim. Coding was conducted by two researchers, in consultation with a third researcher on the team. Initial coding of the data generated themes, which were included in a second round of coding [34]. All members of the research team discussed the coding of the data and assisted with establishing relationships between themes and constructing hierarchies. Data analysis was conducted quantitatively and qualitatively. Approval was obtained from the Stellenbosch University Health Research Ethics Committee (Ref N11/07/227) as well as from the University of Pretoria Research Ethics Committee (Ref s190/2011). All participants in this study consented voluntarily.

## Results

In total 212 participants were approached to participate in this biological samples study and 200/212 agreed yielding a response rate of 94%.

The average age of the participants was 48 years, ranging from 18 to 81 years. The majority of participants (148/200 = 74%) were female. Most participants (81%) had received a high school education (grade 8 to 12). Some (10.5%) had only received primary school education. A few participants had not received any schooling (2%) and a minority of participants had received tertiary education (6.5%). Participants belonged to various religious denominations with the majority being Christian (92.5%). The main home language of participants was as follows: Afrikaans (41%), Xhosa (17%) and English (14%). Most participants were able to read a newspaper in English (87.5%) or Afrikaans (63%) and all participants were happy to conduct the interview in English or Afrikaans.

While most participants (155/200 = 77.5%) felt they had a choice in terms of providing samples for the research project in which they were enrolled, as expressed in the following responses:

I wanted to give [blood] because it was for a good reason to help others.

I gave [blood] willingly for my own good.

41/200 (20.5%) indicated that they had no choice as implied by the responses below:

They just said they have to take my blood.

The doctor just said I need a sample for this or that.

I was instructed to give the sample.

### Storage of samples

Participants were asked how they felt about the possibility that their specimens could be stored after the initial round of tests had been conducted. Most research participants (155/200 = 77.5%) were comfortable with the idea of sample storage. The most commonly cited reason was that once the sample had been donated, they would have no use for it and storage would not affect them. However, 24/200 (12%) indicated that they would want reasons for storage and would want to give permission for storage:

It’s my blood so I would want to know where and why it is being stored.

I would like to be informed if they want to store my blood.

### Future use of specimens and consent

Despite most participants voicing no objection to storage of their samples, almost half the sample (99/200) (49.5%) indicated that they would want to be contacted each time the sample was re-used. Even after the option of a research ethics committee consenting on their behalf was explained in detail, participants were not in favour of this option. Almost half the sample would want to be re-contacted to give consent, the other half would allow a REC to consent on their behalf.

### Export of specimens

While most participants (151/200 = 75%) did not object to exportation of samples, 25/200 (10%) expressed strong concerns and objections. These participants expressed their concerns as follows:

Some countries would maybe use it for satanic rituals.

Not to the African countries. You hear a lot of stories like they use the blood for their cultural things.

As long as it’s not Zimbabwe. They're going to mix my blood with Mugabe.

[Blood must be used] only in Africa. I don’t want it to go out of Africa.

[Blood] must not be taken to the United States, European countries and UK.

As long as it’s not Israel – Israel is an open enemy of the Muslim community.

[Not] Somalia because its far away. I don’t want my samples going very far.

### Benefit sharing

A substantial proportion of participants in this survey (39.5%) indicated that they would mind if researchers or research organisations generated profits from the research in which they were involved. Of this subgroup, 34/79 (43%) expressed a desire for a share of the profits while 45/79 (56%) indicated that they would be very unhappy. A further 19.5% of participants would not mind if profits were generated provided that research was conducted for a good cause.

They must give me a portion of the profit because it is my blood.

It’s funny because the company is using my child’s blood. They must share the money.

I will sue them because I feel it’s a criminal offence

Would be unhappy - how can they make a profit?

## Discussion

The empirical data generated by our study reflects interesting participant perspectives on sample storage, export, re-use and benefit sharing in the research process. Underlying many of these perspectives is the concept of ownership of biological samples. Although participants indicated support for sample collection and storage, which is consistent with existing literature [32,33,35-37], they also expressed strong views about storage and future use, export and benefit sharing.

Our data suggest that only half the participants were in favour of a one-time broad consent procedure when donating samples for storage and re-use, while half felt that re-consent should be sought for future studies and that an REC could not decide on their behalf. This is in stark contrast to the findings of Wendler *et al.* in Uganda, who reported that 95% of participants would be happy not to be re-contacted, as long as the re-use was approved by an Institutional Review Board. Similarly, Chen and Pentz found that most participants would authorize unlimited future research [36,37]. Our findings are similar to the Abou Zeid study in Egypt where 44.3% of the 600 participants surveyed preferred consent options regarding storage and 39% of this subsection desired the consent option for future research to be restricted to the illness under study. Likewise, the study by Igbe *et al.* in Nigeria revealed that half the participants interviewed were in favour of broad consent, while 25% favoured restricted consent and 25% preferred a tiered consent process. Given participant perspectives on future use of samples in African settings, it is important to allow participants to express their choice regarding consent for secondary use of samples.

The Declaration of Helsinki [38] makes provision for consent by a research ethics committee in paragraph 25:

For medical research using identifiable human material or data, physicians must normally seek consent for the collection, analysis, storage and/or re-use. There may be situations where consent would be impossible or impractical to obtain for such research or would pose a threat to the validity of the research. In such situations the research may be done only after consideration and approval of a research ethics committee.

Half our participants did not support such substituted decision making. This finding would suggest that relying on ethics committees to make re-use decisions can be challenging in a community where the focus of participants’ trust is more specifically on actual researchers than the REC itself [31]. It is possible that education of participants about the role of RECs has the potential to change this perception.

Similar to the study by Van Schalkwyk, 19.5% of participants had no desire to share in profits if research was conducted for a good cause [31]. However, 39.5% of participants would mind if a profit was made. Of this subgroup, 43% expressed a desire for a share of the profits while 56% would be very unhappy. This finding is lower than the Abou-Zeid study where 32.8% of participants desired a share in any commercial profits [12]. This understanding of benefit sharing is a matter for concern given that most consent forms relating to biological sample collection and genetic testing contain a paragraph that indicates the following:

“Discoveries made with your DNA samples may be patented by us and the university. These patents may be sold or licensed, which could give a company the sole right to make and sell products or offer testing based on discovery. Royalties may be paid to us, the university and the sponsor. It is not our intent to share any of these possible royalties with you.” [39].

Despite signing consent forms with a similar provision, research participants appear not to fully comprehend this limitation in terms of benefit sharing. For participants, benefit sharing could range from monetary returns, to access to interventions and/or health care. The Declaration of Helsinki encourages comprehensive benefit sharing that is not limited to access to experimental interventions:

• 33. At the conclusion of the study, patients entered into the study are entitled to be informed about the outcome of the study and to share any benefits that result from it, for example, access to interventions identified as beneficial in the study or to other appropriate care or benefits.

The disconnect between what consent documents may indicate, what Helsinki aspires towards and what participants actually expect needs to be carefully considered by researchers and REC members alike.

While most participants expressed a sense of comfort with exportation of samples from South Africa, strong views emerged from 10% of respondents. These views reflected a sense of discomfort with respect to exportation of samples to other African countries as well as to developed countries like the United States, Europe and the United Kingdom. Similar views were expressed in the Abou-Zeid study where 62% of participants expressed their desire to export samples but only to other Arab countries [12]. This issue reflects the potential for further research and has implications for the wording of consent forms where exportation of samples is discussed.

Finally, the concept of ownership of samples is a fundamental question that needs to be explored further. While some participants discussed samples in the context of a donation, many still retained use of the phrase “my blood” or “my child’s blood”. Participants in our survey clearly expressed a sense of “ownership” of their samples, as is implied by the repeated references to “my blood”. This is contrary to the concept of donation of samples in the context of research. Such perspectives were related to the need to be re-contacted for consent for future use and with respect to benefit sharing. It is therefore critical that ownership issues are clarified with communities as part of the community engagement process and with research participants during recruitment and that this concept is revisited and reinforced during the consent process.

This study is not without limitations. Conducting research of this nature is challenging in several respects. In order to gain access to research participants on actual study sites, it is important that research teams do not feel threatened. We had to clarify that the purpose of our study was not to assess the consent process at the sites but rather to obtain participant views on biological sample collection. As such, site selection was limited to those sites where the principal investigator did not object to our line of questioning. We selected research participants at those sites where biological sample collection was part of the research process. However, we were not able to compare the information given in consent forms at various sites regarding sample collection with the understanding of that information by research participants.

## Conclusion

The empirical findings of this study serve to highlight the fact that participants display a wide and complex range of views regarding the use of biological samples in research. Participants hold strong views on future use, export and benefit sharing and a consent process that allows for choice with respect to future use and export of samples seems prudent. Further research needs to be conducted to explore the concept of ownership of samples and benefit sharing in different communities, both in South Africa and other resource constrained countries, to improve the consent process and to respect participant autonomy. Community engagement to clarify understanding of these concepts is critical. However, the views of researchers must also be elicited and balanced with participant views in the best interests of science and society. Research ethics committees have an important role to play in assessing consent forms and processes to ensure that information relating to biological sample collection and use is stated in a clear and unambiguous manner.

## Competing interests

The authors declare that they have no competing interests.

## Authors’ contributions

KM conceptualized the research project, conducted the literature review, supervised and co-ordinated data collection at the Western Cape site and prepared the first draft of this paper. NS, KM and TR designed the questionnaire and standardized it so that it could be used across 2 sites. NS conducted an independent literature review, identified a research site in Pretoria and collected and analysed the empirical data at the Pretoria site. TR supervised the study at the Pretoria site, assisted with data analysis and commented on drafts of this paper. KF assisted with the literature review, conducted interviews at the Western Cape site, completed data capture and edited drafts of the article. All authors read and approve the final manuscript.

## Pre-publication history

The pre-publication history for this paper can be accessed here:

http://www.biomedcentral.com/1472-6939/15/4/prepub
